# Modified FCI (Fédération Cynologique Internationale) Scoring of the Coxofemoral Joint in Labrador Retrievers Without and With Hip Dysplasia

**DOI:** 10.3389/fvets.2022.800237

**Published:** 2022-03-18

**Authors:** Ayman A. Mostafa, Menna A. Nahla, Khaled M. Ali, Clifford R. Berry

**Affiliations:** ^1^Department of Small Animal Surgery and Radiology, Faculty of Veterinary Medicine, Cairo University, Giza, Egypt; ^2^Diagnostic Imaging, Department of MBS, College of Veterinary Medicine, North Carolina State University, Raleigh, NC, United States

**Keywords:** FCI score, coxofemoral, acetabular femoral head coverage, hip dysplasia, labrador

## Abstract

The objective is to propose a modified FCI scoring protocol of the canine hip joint *via*: (1) providing morphometric criteria of each score; (2) quantifying the extent of lateral and dorsal acetabular femoral head (AFH) coverage; (3) evaluating the steepness of cranial acetabular edge (acetabular index angle) and inclination angle (IA) in normal and dysplastic coxofemoral joints of Labrador Retrievers. The long-term goal is to achieve a selective breeding protocol using parental phenotypically healthy coxofemoral joints based on the standard extended-leg VD radiograph to help reduce the prevalence of CHD among offspring. Investigated populations were classified into normal (grade A) and dysplastic coxofemoral joints (grades B to E) based on the morphometric criteria previously established by the conventional FCI scoring system. Center-edge (CE) angle, Norberg angle (NA), indices of dorsal AFH coverage width and area, acetabular index angle, and inclination angle were determined for each group. Variables were compared between groups using ANOVA. Spearman correlation coefficient was used to determine the linear relationship between selected variables. Overall, all radiographic measurements differed significantly (*P* < 0.0001) among the five tested groups using ANOVA test. Dorsal AFH coverage area index was the only measure that differed significantly (*P* ≤ 0.007) between every two consecutive groups using Tukey's test. Significant correlations were identified between the Norberg and CE angles (*r*_s_ = 0.95, *P* < 0.0001), the width and area of dorsal AFH coverage (*r*_*s*_ = 0.96, *P* < 0.0001), and the radiographic techniques utilized to assess lateral vs. dorsal AFH coverage (*r*_*s*_ ≥ 0.80, *P* < 0.0001). Evaluation of CE-angle, dorsal AFH coverage area index and acetabular index angle is recommended during selective breeding to include parents with radiographically healthy joints and reduce the incidence of hip dysplasia among offspring. Dogs with CE-angle <27°, dorsal AFH coverage area index <53%, and/or acetabular index angle >9° may be consistent with hip dysplasia and are recommended to be excluded from potential breeding groups. Re-evaluation of coxofemoral joints with borderline values located between near-normal and mildly dysplastic coxofemoral joints is strongly recommended to be performed after 6 months.

## Introduction

Canine hip dysplasia (CHD) is a developmental, heritable and multifactorial disorder of the coxofemoral joint with associated joint laxity and incongruity that predisposes to osteoarthritis (1–3). It affects mainly rapidly growing large breed dogs (4), such as German Shepherds, Labrador Retrievers, and Boxers (5, 6). Extended-leg ventrodorsal (VD) pelvic radiograph, first introduced in the 1960s by Riser (7), remains the most commonly used technique for evaluating canine coxofemoral joint according to the FCI (Fédération Cynologique Internationale), OFA (Orthopedic Foundation for Animals), and BVA/KC (British Veterinary Association and the Kennel Club) (8). Various radiographic measurements have been used to evaluate hip dysplasia in adult humans and dogs (9–12). Norberg angle (NA) is a common radiographic measure utilized to assess the degree of lateral acetabular femoral head (AFH) coverage in dogs (13, 14). However, the technique of measuring the NA relies basically on consideration of both coxofemoral joints. In the human literature, Center-edge (CE) and acetabular index/slope angles were previously utilized to quantify the degree of lateral AFH coverage and measure the steepness of acetabular roof of each hip joint separately on the anteroposterior (AP) view, respectively (9, 10, 15, 16). To our knowledge, two veterinary reports have measured CE (12, 17) and acetabular index (12) angles on extended-leg VD radiographs. However, these studies relied on the contralateral hip joint to measure the CE angle by using the long axis of the pelvis (the axis perpendicular to a line connecting FH centers) (17) or *via* using the “mid-sagittal” axis (12). Acetabular index angle is a measurement for the steepness of the weight-bearing surface of the cranial acetabulum in humans (10) and dogs (12). In these two studies, the acetabular index angle relied on the use of transverse planes drawn in the supine position of humans and dorsal recumbency of dogs. Unlike a human AP pelvic radiograph that is taken in a supine or standing position, the use of a mid-sagittal or transverse axis on canine VD pelvic radiographs are obtained with the dog in dorsal recumbency and thereby may not be efficient to precisely measure the CE or acetabular index angle. Furthermore, the previously calculated CE and acetabular index angles in veterinary literature relied on both right and left coxofemoral joints using a line connecting the corresponding FH centers. The current study is the first to measure the CE and acetabular index angles for each hip joint separately (independent angles) and to quantify the degree of dorsal AFH coverage and inclination angle in Labrador Retrievers without and with hip dysplasia and secondary osteoarthritis. The impetus of the present study was the observation that the current selective breeding protocols were not potentially effective enough to markedly reduce CHD numbers using the current conventional radiographic procedures, such as Norberg angle (18–20).

Therefore, our main objective is to create a modified FCI scoring system of the canine hip joint *via* providing morphometric criteria for each score and quantifying the extent of lateral and dorsal AFH coverage, the steepness of cranial acetabular edge (acetabular index angle), and inclination angle in normal and dysplastic coxofemoral joints of Labrador Retrievers. Our hypothesis is that AFH coverage, cranial acetabular edge steepness, and angle of inclination would vary significantly in dysplastic hip joints, as well as with the grade of the disease, compared to those calculated for normal or near-normal joints. Our long-term goal is to achieve a selective breeding protocol using parental phenotypically healthy coxofemoral joints based on the standard extended-leg VD radiograph to help reduce the prevalence of CHD among offspring in the future.

## Materials and Methods

### Population

The Scientific Committee of the Department of Surgery and Radiology at the Faculty of Veterinary Medicine, Cairo University approved the retrospective study protocol before investigation. Medical records and extended VD pelvic radiographs of Labrador Retrievers with normal and dysplastic hip joints were retrieved from the database of the Small Animal Hospitals at Cairo University and University of Florida, Colleges of Veterinary Medicine from May 2005 to November 2020. All digitized radiographs were approved in terms of quality and positioning (21, 22), and were then categorized into five groups (A to E) by board-certified (CB) and qualified (AM) radiologists. The five groups categorization was performed based on the morphometric criteria previously established by the conventional FCI scoring protocol of CHD (8, 23, 24). The five groups included one normal (grade A), one near-normal (grade B), and three dysplastic (grades C–E) groups. The three dysplastic groups included mildly (grade C), moderately (grade D), and severely (grade E) dysplastic joints. Grade A (normal joint) included a coxofemoral joint with narrow space and sharply margined, perfectly parallel articular surfaces (perfectly congruent joint). The near-normal hip (grade B) included a coxofemoral joint with sharply margined, non-parallel articular surfaces with associated slightly widened joint space (minimal joint incongruence). The hip joint was considered mildly dysplastic (grade C) if there were incongruity of the coxofemoral joint (wedged-shaped joint space) and slight flattening of the craniolateral acetabular rim. Moderately dysplastic hip (grade D) exhibited obvious joint incongruity with subluxation and flattening of the craniolateral acetabular rim. Severely dysplastic hip (grade E) exhibited coxofemoral luxation or distinct subluxation and flattening and deformity of the femoral head. According to previous studies, coxofemoral joints with a Norberg angle ≥105° were considered normal or near normal; whereas, those with a NA < 105° were considered dysplastic (1, 22, 25, 26). Radiographic existence of coxofemoral osteoarthritis were recorded in our modified FCI hip scoring.

### Radiographic Measurements

All radiographic measurements were performed on digitized radiographs by the same investigator (MN) who was unaware of the status of the sorted groups. The measurements were made using medical and radiologic image processing software (ImageJ 1.41/Java 1.6.0_21) with a magnification of 200 (13, 14). Six radiographic parameters were evaluated for each coxofemoral joint. Center-edge (CE) and Norberg angles (NA) were measured to evaluate the degree of lateral acetabular femoral head (AFH) coverage. Indices of dorsal AFH coverage width and area were calculated to determine the extent of dorsal AFH coverage. The acetabular index/slope angle was measured to quantify the steepness of the cranial acetabular edge (acetabular “sourcil” slope). The inclination angle evaluated the proximodistal alignment of the femoral head and neck relative to the corresponding femoral axis.

**Figure 1:**
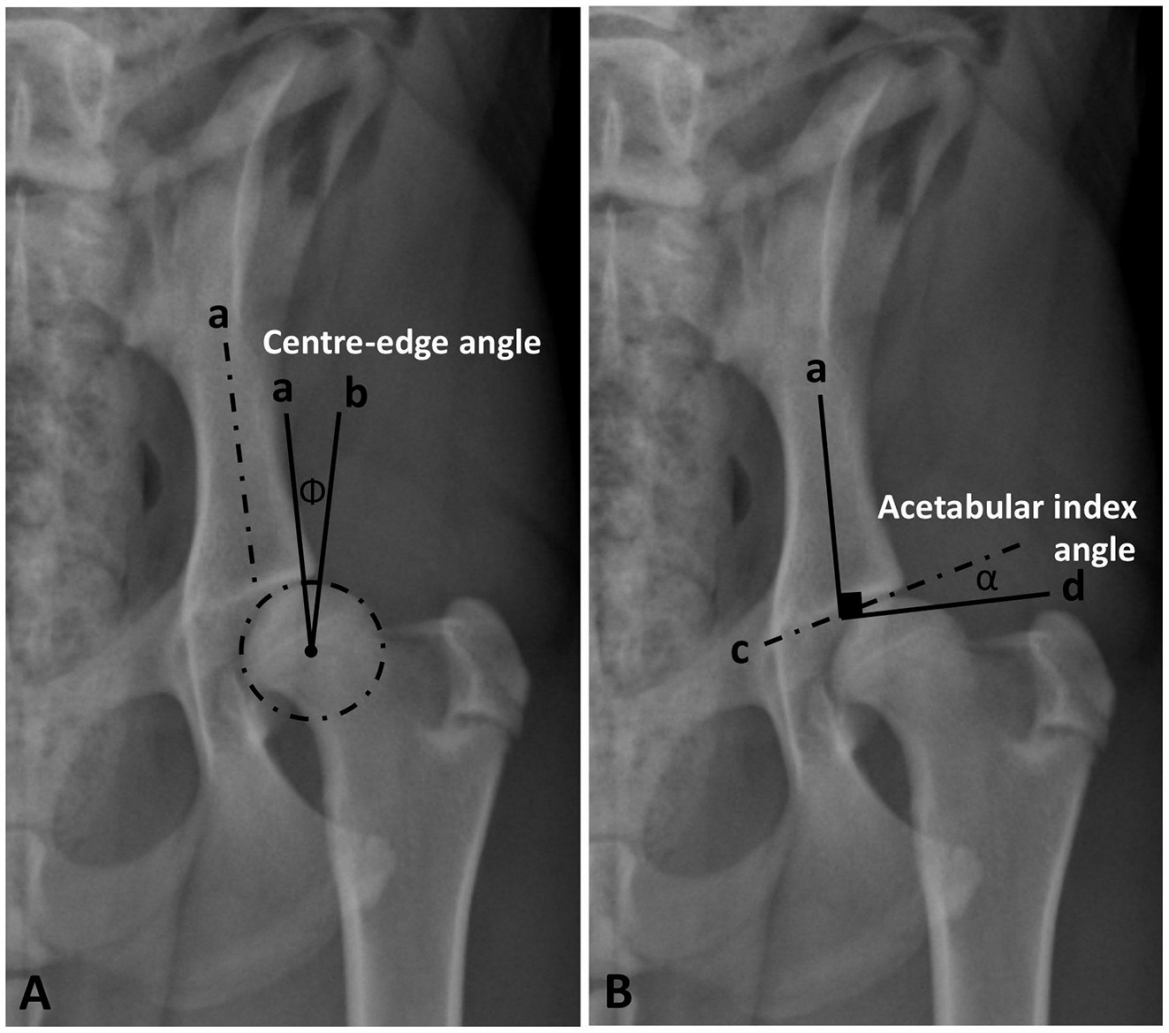
Ventrodorsal pelvic radiograph of a mildly dysplastic coxofemoral joint illustrating measurements of Center-edge angle (Φ) **(A)** and acetabular index/slope angle (α) **(B)**. *a*, long axis of the iliac body; *b*, a line originating from the femoral head center and tangential to lateral acetabular rim; *c*, a line tangential to lateral and medial extents of the cranial acetabular rim (acetabular sourcil); *d*, a horizontal line perpendicular to the iliac axis (*a*).

Initially, a best fit circle outlining the femoral head was drawn to define its center and calculate its area. Norberg and inclination angles were measured according to the previously published veterinary literature (13, 14, 27–29). Center-edge (CE) and acetabular index angles were modified from the previously established human procedures (9, 10, 15, 16, 30). The CE angle was measured between two straight lines originating from the center of the femoral head, a line tangential to the lateral acetabular rim and a second line parallel to the longitudinal axis of the body of the corresponding ilium (iliac axis) (14) (Figure 1A). The acetabular index/slope angle formed between a line connecting the lateral and medial extents of the sclerotic cranial acetabular edge (acetabular sourcil slope) and a horizontal line perpendicular to the corresponding iliac axis (Figure 1B).

**Figure 2:**
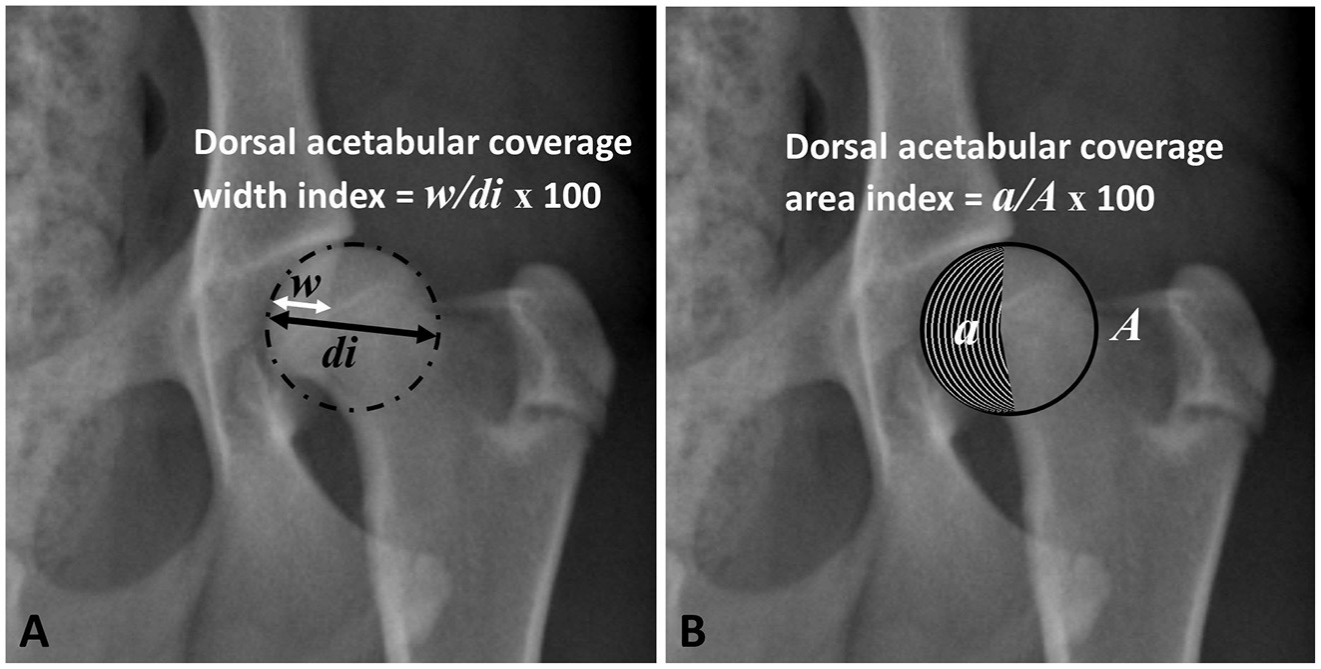
Ventrodorsal pelvic radiograph of a mildly dysplastic coxofemoral joint illustrating measurements of dorsal acetabular femoral head coverage width index **(A)** and dorsal acetabular coverage area index **(B)**. *w*, width of dorsal acetabular coverage; *di*, diameter of the femoral head; *a*, area of dorsal acetabular coverage; *A*, area of the femoral head.

Femoral head (FH) diameter was drawn perpendicular to and bisecting the corresponding dorsal acetabular edge to measure the width of the dorsal acetabulum that overlays the FH at this level (Figure 2A). The index of dorsal AFH coverage width was then calculated by dividing the width of dorsal acetabular coverage by FH diameter (Figure 2A). The index of dorsal acetabular coverage area was calculated by dividing the area of the femoral head that is covered by the corresponding dorsal acetabulum and bounded laterally by the dorsal acetabular edge by the overall femoral head area (Figure 2B).

### Statistical Analysis

The data were analyzed using commercially available statistical software (Graph-Pad Prism^®^ version 8.00, La Jolla, California, USA). All data were tested for normality using the Kolmogorov-Smirnov test and were proven to be normally distributed. A significance level of *P* < 0.05 was set. Mean (±SD) values of all parameters were calculated, and variables of interest were compared between the five tested groups (A–E) using ANOVA test. A 95% confidence interval was calculated for selected measurements. A Spearman rank correlation coefficient (*r*_*s*_) was calculated to determine the linear relationship between selected variables.

## Results

### Population

A total of 175 purebred Labrador Retrievers (337 hip joints) with radiographically normal (grade A, 111 joints “32.9%”), near normal (grade B, 38 joints “11.3%”), mildly dysplastic (grade C, 46 joints “13.6%”), moderately dysplastic (grade D, 67 joints “19.9%”) and severely dysplastic (grade E, 75 joints “22.3%”) coxofemoral joints were identified. There were 45 coxofemoral joints (13.4%) that exhibited secondary coxarthrosis (35 severely, 9 moderately, and 1 mildly dysplastic joints). Subluxation was identified in 46 (13.6%) coxofemoral joints (37 severely and 9 moderately dysplastic joints). The completely luxated coxofemoral joints (13 joints, 3.9%) were excluded from the radiographic measurements. A Morgan line was identified in 68 (20.2%) coxofemoral joints (12 near normal joints and 21 mildly, 18 moderately, and 17 severely dysplastic joints). There was a significant difference (*P* < 0.0001) in the age between groups; however, body weight did not differ (*P* = 0.5) between groups (Table 1). There were 100 males (23 neutered) and 75 females (21 neutered) enrolled in the present study, with an overall female to male ratio being 1:1.3.

**Table 1:** Radiographic measurements and age and body weight assessment.

**Variable**	**Normal hip joints**		**Near normal hip joints**		**Mild hip dysplasia**		**Moderate hip dysplasia**		**Severe hip dysplasia**		**ANOVA**
	**(*****n*** **=** **111)**		**(*****n*** **=** **38)**		**(*****n*** **=** **46)**		**(*****n*** **=** **67)**		**(*****n*** **=** **75)**		**(*P*-value)**
	**Mean ±SD**	**95% CI**	**Mean ±SD**	**95% CI**	**Mean ±SD**	**95% CI**	**Mean ±SD**	**95% CI**	**Mean ±SD**	**95% CI**	
Age/y	9.1 ± 2.8	8.4–9.8	8.4 ± 2.2	7.5–9.3	7.1 ± 3.1	6.1–8.2	5.6 ± 3.9	4.5–6.6	5.7 ± 4.0	4.6–6.8	<0.0001
Body weight/kg	33.1 ± 6.5	31.6–34.7	32.6 ± 5.4	30.7–34.6	34.5 ± 5.0	31.7–37.3	32.8 ± 6.7	30.5–35.1	31.9 ± 6.2	29.8–34.0	0.5301
Norberg angle (degree)	109.9 ± 3.2	109.3–110.5	109.0 ± 2.9	108.0–109.9	102.0 ± 1.5	101.5–102.4	94.8 ± 2.8	94.1–95.5	81.2 ± 7.6	79.4–83.0	<0.0001
Center-edge angle (degree)	28.1 ± 3.6	27.5–28.8	27.7 ± 3.6	26.5–28.9	20.4 ± 3.7	19.3–21.5	12.7 ± 3.8	11.8–13.7	−0.48 ± 8.1	−2.4–1.4	
Dorsal AFH coverage width index	0.56 ± 0.05	0.55–0.57	0.53 ± 0.06	0.52–0.55	0.47 ± 0.06	0.45–0.49	0.39 ± 0.09	0.36–0.41	0.28 ± 0.09	0.26–0.30	
Dorsal AFH coverage area index	0.60 ± 0.05	0.59–0.61	0.55 ± 0.06	0.53–0.57	0.49 ± 0.08	0.47–0.51	0.36 ± 0.10	0.33–0.38	0.24 ± 0.10	0.21–0.26	
Acetabular index angle (degree)	8.6 ± 4.1	7.8–9.3	7.8 ± 3.3	6.7–8.9	11.1 ± 3.9	9.9–12.3	16.7 ± 8.1	14.7–18.7	27.8 ± 18.4	23.6–32.1	
Inclination angle (degree)	130.6 ± 5.8	129.5–131.7	135.7 ± 6.1	133.6 ± 137.7	131.1 ± 7.4	128.9–133.3	127.0 ± 7.6	125.1–128.9	124.1 ± 10.5	121.7–126.6	

*The mean (±SD) values and 95% CIs for the age and body weight, as well as for the radiographic measurements quantifying lateral AFH coverage (Norberg and Center-edge angles), dorsal AFH coverage (width and area indices), steepness of cranial acetabular edge (acetabular index angle), and proximodistal alignment of femoral head and neck relative to the femoral axis (inclination angle) for 175 Labrador Retrievers with normal and dysplastic coxofemoral joints*.

*Significant value was set at a p < 0.05*.

*SD, standard deviation; CIs, confidence intervals; AFH, acetabular femoral head*.

### Radiographic Measurements

**Figure 3:**
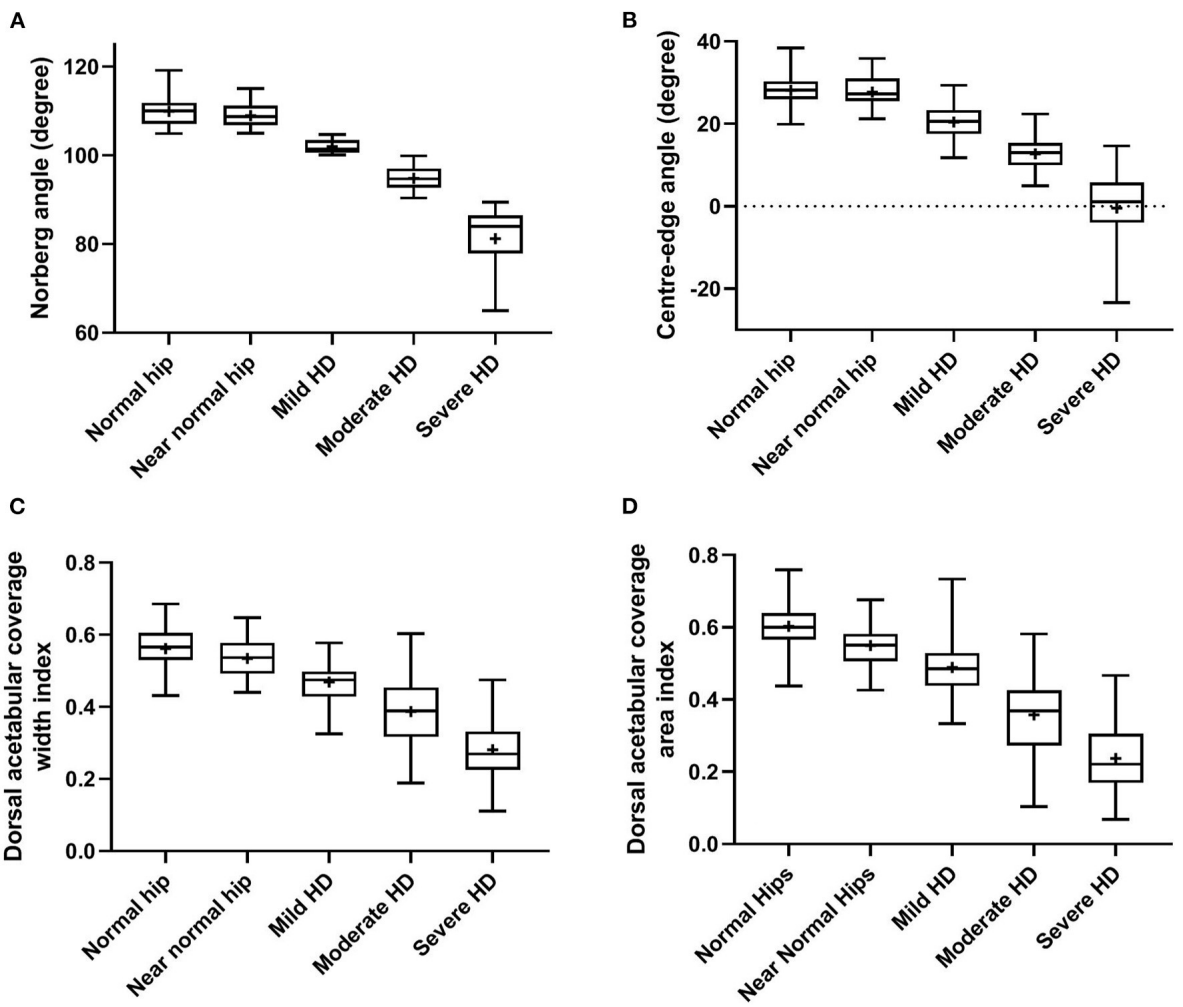
Box-and-whisker plots of Norberg angle **(A)**, Center-edge angle **(B)**, dorsal acetabular coverage width index **(C)**, and dorsal acetabular coverage area index **(D)** for normal, near-normal, mildly dysplastic, moderately dysplastic, and severely dysplastic coxofemoral joints.

**Figure 4:**
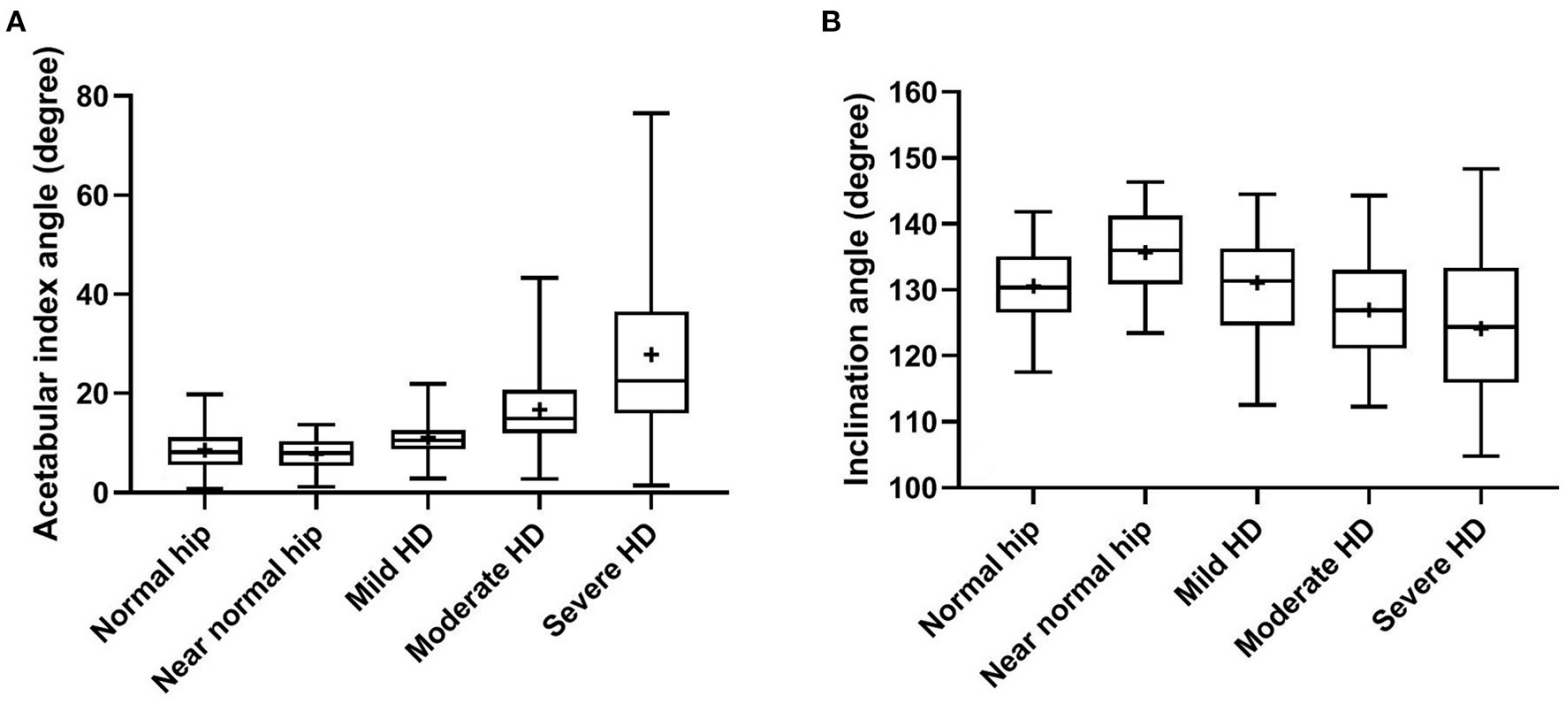
Box-and-whisker plots of acetabular index angle **(A)** and inclination angle **(B)** for normal, near-normal, mildly dysplastic, moderately dysplastic, and severely dysplastic coxofemoral joints.

Statistically significant differences (*P* < 0.0001, ANOVA test) were evident in all reported radiographic measurements among the five tested groups (Table 1). However, there was no significant difference (*P* ≥ 0.80, Tukey's test) between normal and near-normal coxofemoral joints in the measurements utilized to quantify lateral AFH coverage (NA and CE-angle) (Figures 3A,B). Although dorsal acetabular area index showed a better femoral head coverage in normal compared to near-normal coxofemoral joints (*P* = 0.005, Tukey's test) (Figure 3D), dorsal AFH width index did not differ between normal and near-normal joints (*P* ≥ 0.25, Tukey's test) (Figure 3C). No significant difference (*P* ≥ 0.55) was identified in the acetabular index angle between normal, near normal, and mildly dysplastic joints (Table 1, Figure 4A). The decreased angle of inclination (coxa-vara) was consistent with moderate and severe hip dysplasia. However, inclination angle did not differ significantly between mildly dysplastic hip and each of normal and near normal coxofemoral joints (*P* ≥ 0.052), and between moderately and severely dysplastic hip joints (*P* = 0.200) (Figure 4B). Mean (±SD) values and 95% CIs of all reported radiographic measurements are summarized in Table 1.

**Figure 5:**
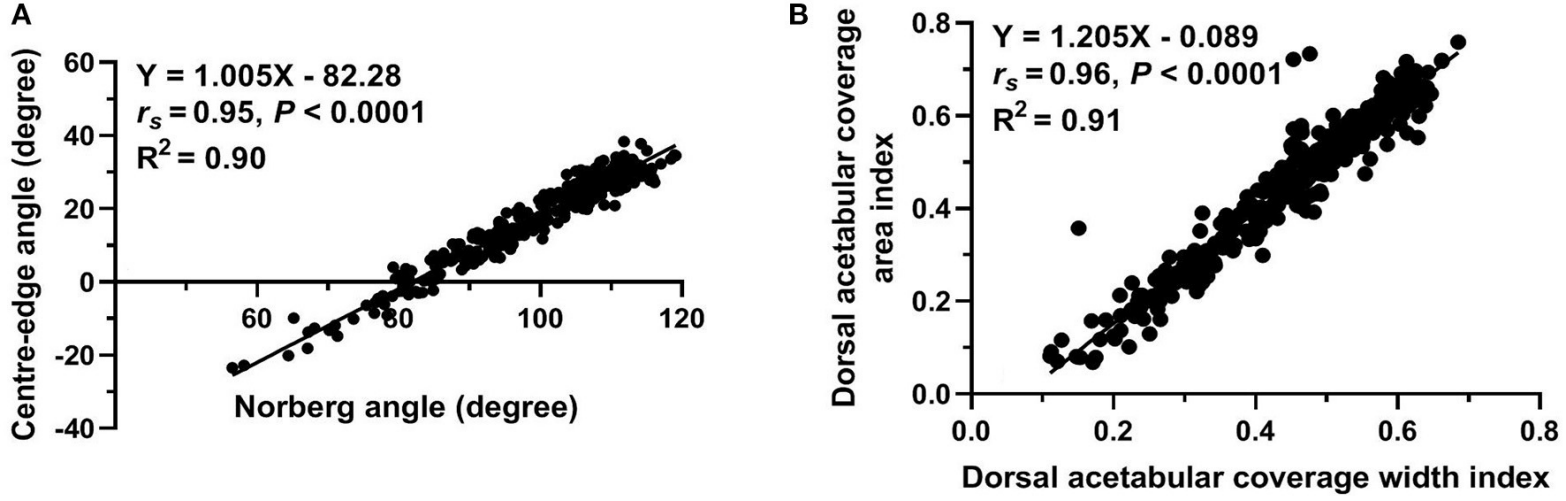
Scatterplots of Center-edge angle vs. Norberg angle **(A)** and dorsal acetabular coverage area index vs. dorsal acetabular coverage width index **(B)** determined for 337 normal and dysplastic coxofemoral joints of 175 Labrador Retrievers.

**Figure 6:**
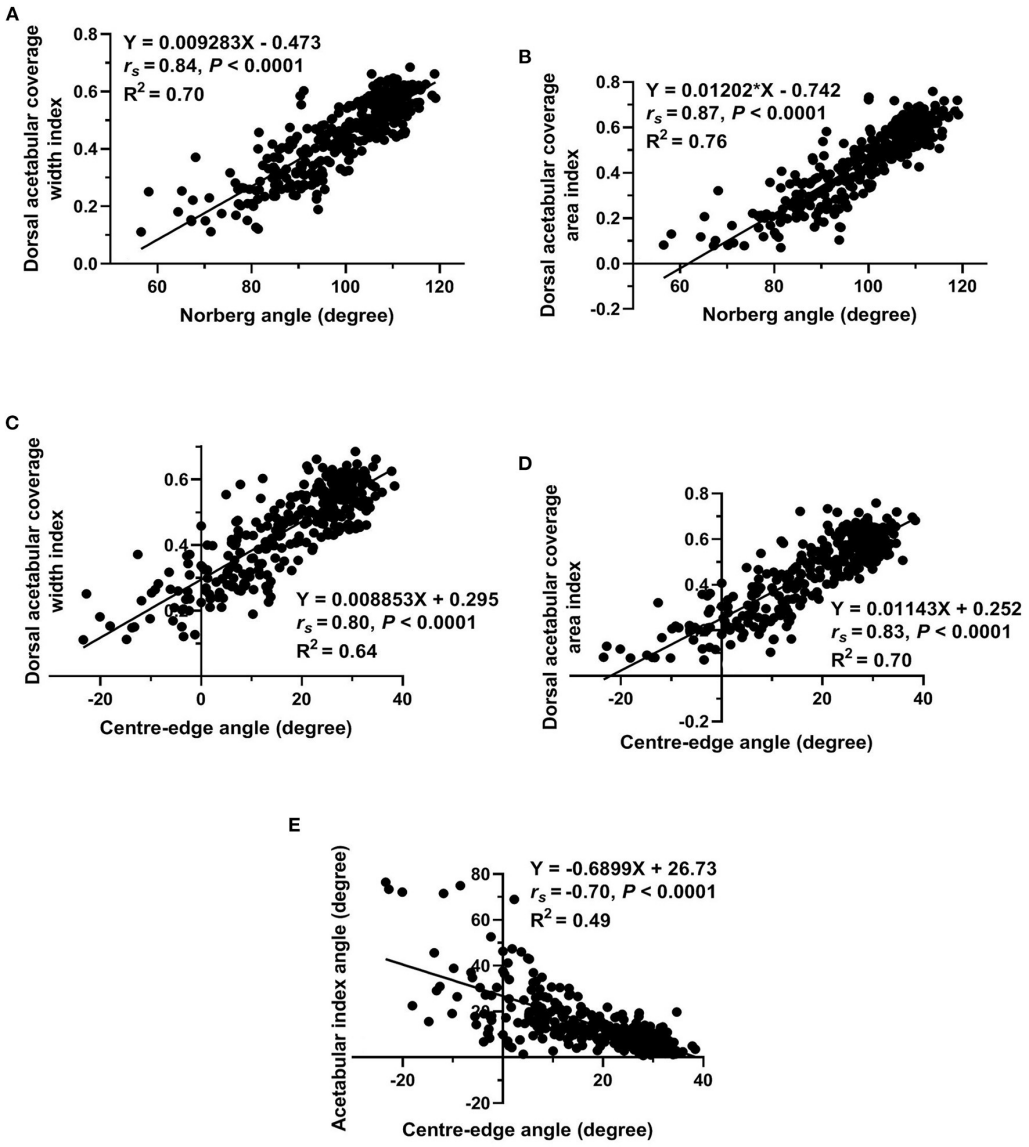
Scatterplots of Norberg angle vs. each of dorsal acetabular coverage width index and dorsal acetabular coverage area index **(A,B)**, of Center-edge angle vs. each of dorsal acetabular coverage width index and dorsal acetabular coverage area index **(C,D)**, and of acetabular index angle vs. Center-edge angle **(E)** determined for 337 normal and dysplastic coxofemoral joints of 175 Labrador Retrievers.

Regarding measurements used to evaluate lateral AFH coverage, a strong positive correlation (*r*_s_ = 0.95, *P* < 0.0001) was identified between Norberg and Center-edge angles (Figure 5A). Furthermore, there was a strong positive correlation (*r*_*s*_ = 0.96, *P* < 0.0001) between the two indices (width and area) utilized to assess the degree of dorsal AFH coverage (Figure 5B). Strong positive correlations were determined between the radiographic techniques used to assess lateral vs. dorsal AFH coverage (*r*_*s*_ ≥ 0.80, *P* < 0.0001) (Figures 6A–D). A strong negative correlation (*r*_s_ = −0.70, *P* < 0.0001) was identified between acetabular index angle and Center-edge (CE) angle (Figure 6E). Significant weak correlations (*r*_*s*_ ≤ 0.38, P < 0.0001) were identified between the inclination angle and each of the radiographic measurements reported in the present study. According to the morphometric criteria and the results of the radiographic measurements associated with each tested group, a modified FCI scoring system was created (Table 2).

**Table 2:** Modified FCI scoring protocol of the coxofemoral joint of labrador retrievers.

**Grade**		**Morphometric criteria**	**Reference values of radiographic measurements**
**A**	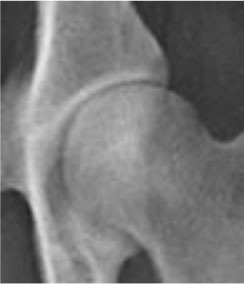	- Perfectly congruent joint - Joint space appears narrow with sharply margined and perfectly parallel articular margins	- Dorsal acetabular femoral head coverage area index ≥ 59 % - Center-edge angle ≥ 27° - Acetabular index angle ≤ 9° - Norberg angle ≥ 109° - Dorsal acetabular femoral head coverage width index ≥ 55%
**B**	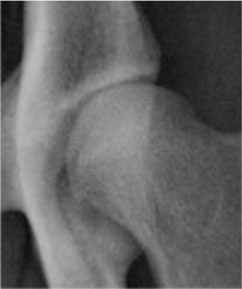	- Minimal joint incongruence - Joint space appears slightly widened with non-parallel coxofemoral articular margins - Morgan line may be noted	- Dorsal acetabular femoral head coverage area index (53–57%) - Center-edge angle ≥ 27° - Acetabular index angle ≤ 9° - Norberg angle ≥ 109° - Dorsal acetabular femoral head coverage width index (52–55%)
**C**	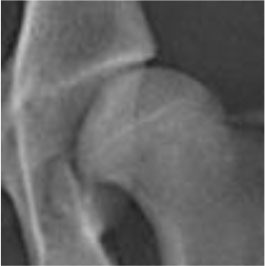	- Incongruity of the joint (wedged-shape joint space) - Flattening of the craniolateral acetabular rim may be present - Minimal signs of osteoarthritis may be noted - Morgan line may be noted	- Dorsal acetabular femoral head coverage area index (47–51%) - Center-edge angle **(**19.3–21.5°) - Acetabular index angle **(**9.9–12.3°) - Norberg angle (101.5–102.4°) - Dorsal acetabular femoral head coverage width index (45–49%)
**D**	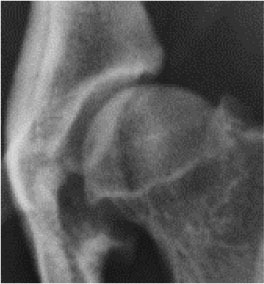	- Obvious incongruity of the joint - Subluxation may be present - Signs of osteoarthritis - Morgan line may be noted - Flattening of the craniolateral acetabular rim. - Deformity of the femoral head may be present	- Dorsal acetabular femoral head coverage area index (33–38%) - Center-edge angle (11.8–13.7°) - Acetabular index angle (14.7–18.7°) - Norberg angle (94.1–95.5°) - Dorsal acetabular femoral head coverage width index (36–41%)
**E**	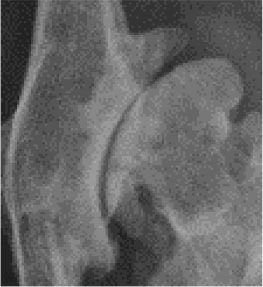	- Luxation or distinct subluxations are present. - Severe deformity of the femoral head (mushroom-shaped and flattened) - Signs of osteoarthritis - Morgan line may be noted	- Dorsal acetabular femoral head coverage area index ≤ 26% - Center-edge angle ≤ 1.4 - Acetabular index angle ≥ 23.6° - Norberg angle ≤ 83° - Dorsal acetabular femoral head coverage width index ≤ 30%

*The table illustrates the morphometric criteria and reference values of the radiographic measurements of the coxofemoral joint for each investigated score/grade*.

*The gap of values between every two consecutive groups may represent subjects with borderline degrees of hip dysplasia with reevaluation after 6 months being recommended by the authors*.

## Discussion

The main findings of the study reported here were: (1) significant differences in all reported radiographic measurements were evident among the five tested groups. However, Norberg and CE angles did not differ between normal and near-normal hip joints; (2) dorsal AFH coverage width index did not differ between normal and near-normal joints; however, dorsal AFH coverage area index showed better coverage in normal vs. near-normal coxofemoral joints; (3) no difference was identified in the acetabular index angle between normal, near normal, and mildly dysplastic joints; (4) strong correlations were evident between Norberg and Center-edge angles, the width and area indices of dorsal AFH coverage, and between the radiographic techniques utilized to assess lateral vs. dorsal AFH coverage. The strong correlation (*r*_s_ = 0.95) between Norberg and Center-edge angles would support using Center-edge angle as an alternative procedure to quantify lateral AFH coverage without consideration of the contralateral side (CE-angle below 27° may indicate hip dysplasia). The strong correlation between the width and area indices of dorsal AFH coverage would suggest using the area index as an alternative procedure to quantify dorsal AFH coverage (area index below 59% may be consistent with joint incongruence). Acetabular index angle above 9° was consistent with hip dysplasia; (5) coxa-vara was consistent with moderately and severely dysplastic hip joints.

Center-edge (CE) angle and Norberg angle (NA) quantify lateral AFH coverage. Failure of CE-angle and NA to differentiate between normal and near-normal coxofemoral joints may be related to the selected values of NA (≥105°) for near-normal joints that were set in our study based on the conventional FCI criteria (8, 23, 24). This may also explain why the mean NA of our near-normal group (109°) differed from those (105.9 and 105.7°) reported by other two veterinary literatures (3, 17). The means NA of our enrolled coxofemoral joints in group A (109.9°), group C (102°), group D (94.8°), and group E (81.2°) were relatively consistent with the means of the same groups (group A, 108.4–108.8°; group C, 101–102.8°; group D, 94.1–95°; group E, 82–89.4°) reported by previous veterinary literatures (24, 26, 31). However, the means CE-angle of the five tested groups (A, B, C, D, and E) identified in the present study (28.1, 27.7, 20.4, 12.7, −0.48°, respectively) differed from those reported by the previous veterinary literature (16.91, 12.55, 10.65, 6.62, −9.25°, respectively) (24). This discrepancy may be attributed to the modified procedure established in our study to measure the CE-angle *via* utilizing the iliac axis instead of the longitudinal axis used by Meomartino et al.; as well as, the different radiographic projection (DAR view) utilized by the previous study (25). The values of CE-angle reported in our normal and near-normal groups (28°) were relatively consistent with the normal values reported in human literature (25°) (30, 32). Furthermore, recent human literature reported that a hip joint with a CE-angle below 20° was considered dysplastic (33), and this is similar to the value of the CE-angle calculated for our dysplastic joints (≤20.4°) despite of the difference in the anatomy and biomechanics between human and dogs. This may indicate the feasibility of using the iliac axis on canine VD pelvic radiographs instead of utilizing a longitudinal axis which may not be realistic for dogs to perform a radiographic measurement (14, 32). The correlation (*r*_s_= 0.95) identified between NA and CE-angle in the present study was stronger than that (*r*_s_= 0·79) calculated by another veterinary literature (24). Accordingly, the authors would recommend the use of the modified CE-angle over NA to evaluate the degree of lateral AFH coverage of each joint separately without consideration of the contralateral hip joint. This may also aim to overcome the possible imperfection associated with NA previously reported by Doskarova and colleagues (17).

In the present study, a strong positive correlation (*r*_*s*_ = 0.96) was identified between the width and area indices that were calculated to assess the degree of dorsal AFH coverage. A relatively similar correlation (*r*_*s*_ = 0.84) was reported in a previous veterinary study (11). Unlike the width index that failed to differentiate between normal and near normal coxofemoral joints, the area index was able to differentiate between the two groups. Therefore, the authors would recommend using the area index to quantify dorsal AFH coverage during screening dogs before breeding. The ability of the area index to differentiate between normal and near-normal groups may be because the area index determines the overall dorsal AFH coverage area, not just the corresponding width index. Dorsal AFH coverage area index reported in our five tested groups (A–E) (60, 55, 49, 36, 24%, respectively) were relatively consistent with those (59.5, 54.9, 46.3, 32.3, 26.8%, respectively) reported by Tomlinson et al. (26). The median indices of dorsal AFH coverage width and area reported in our near-normal population (54 and 55%, respectively) were relatively consistent with the median values of linear and surface overlap (52 and 54%, respectively) previously reported by a study performed on a wide variety of younger dog breeds (11). This consistency may be due to the quite similarity of the measurement techniques utilized in both studies, regardless of the age or dog breed. However, a better dorsal AFH coverage (57 and 60%, respectively) was identified in our investigated normal coxofemoral joints. Despite the strong correlation (*r*_*s*_ ≥ 0.80) identified between the radiographic parameters utilized to assess lateral vs. dorsal AFH coverage, the authors still recommend doing both measurements to adequately evaluate the overall AFH coverage, especially when evaluating dogs for breeding. This is in agreement with a previous study that recommended evaluation of the percentage of the femoral head covered by the corresponding acetabulum (i.e., dorsolateral subluxation score) to provide more information on the dog's genetic potential (34).

The means acetabular slope angle identified in our five tested groups (A–E) (8.6, 7.8, 11.1, 16.7, 27.8°, respectively) were relatively consistent with those (7.1, 11.6, 11.8, 15.0, 25.2°, respectively) reported by Meomartino et al. (24). The minimal variation in the acetabular slope angle noted between the two studies could be related to the minimal difference between the two used measuring techniques. Failure of the acetabular slope angle to differentiate between normal, near normal, and mildly dysplastic hip joints may indicate that steepness of cranial acetabular edge is most likely evidenced in moderately and severely dysplastic joints. In humans, an acetabular slope angle above 13° (vs. >11° in dogs) was reported to be consistent with hip dysplasia (35). The substantial variation in the standing angle of the hip joint between humans and dogs may result in a variety in the natural load applied on the corresponding acetabulum of each species (14, 36–38). In humans, the natural load applied on the cranial acetabular region may explain the excessive steepness of the cranial acetabular edge previously identified in dysplastic hips (35). However, the natural load applied on the dorsal acetabular region is expected to be greater in dogs than that applied on the cranial acetabular region. This may support the importance of quantifying both dorsal and lateral AFH coverage during the routine screening program of the canine hip joint. The strong negative correlation (*r*_s_ = −0.70, *P* < 0.0001) identified between acetabular slope angle and CE-angle suggests the relative consistency between a low steep acetabular roof and a high lateral AFH coverage in healthy joints. The mean inclination angle of our enrolled normal coxofemoral joints (130.9°) is consistent with the values (129.4°) previously reported in normal large breed dogs (27, 28). In the present study, decreased angle of inclination (coxa-vara) was consistent with moderate and severe hip dysplasia. Nonetheless, the inclination angle failed to differentiate between normal, near normal, and mildly dysplastic coxofemoral joints, as well as between moderately and severely dysplastic joints. In other veterinary literature, the inclination angle showed a non-significant difference between dysplastic and healthy joints (28, 29). This discrepancy may be explained by the feasibility of external femoral rotation, secondary coxarthrosis, and/or coxofemoral subluxation that may influence the angle of inclination (29).

The gap of values noted between every two consecutive groups (Tables 1, 2) may represent individuals with borderline degrees of hip dysplasia, and this may be considered a limitation of the present study. However, the existence of borderline values between different grades of hip dysplasia has also been reported in other veterinary and human literatures (1, 33). Therefore, the authors would recommend comprehensive assessments of both dorsal (width and area indices) and lateral (CE- and Norberg angles) AFH coverage in dogs, especially those with borderline degrees of hip dysplasia. Furthermore, reevaluation of coxofemoral joints with borderline values is strongly recommended to be performed after 6 months, as previously advised by Flückiger (1). The lack of assessment of the reproducibility of the reported radiographic measurements is another limitation of our study, and a future study calculating intra- and inter-observer variability is needed. Furthermore, the study did not investigate the correlation between the clinical signs and the radiographic findings associated with dysplastic coxofemoral joints. However, it has been proven that clinical signs of CHD do not correlate with the severity of the radiographic changes associated with hip joints (39). Besides, our study focused solely on the morphometric criteria and radiographic measurements of normal, near normal, and dysplastic coxofemoral joints (i.e., modified FCI scoring). Lack of assessment of hip joint laxity *via* calculating distraction index (PennHip DI) may limit the efficacy of radiographic determination of AFH coverage in our suggested selective screening protocol, as evaluation of joint laxity would exclude additional individuals from the breeding pool (25). Therefore, a future clinical and radiographic investigation may be warranted on Labrador Retrievers and other dog breeds without and with hip dysplasia. An ongoing similar study has been designed to create a comprehensive screening protocol for German Shepherds' hip joints and to further compare the results with those for Labrador Retrievers.

## Conclusions

Norberg or Center-edge angle below 109 or 27°, respectively, would suggest a lack of optimum lateral AFH coverage and possible joint incongruence. Center-edge angle could be utilized as an alternative to Norberg angle to quantify lateral AFH coverage of each joint separately. Dorsal AFH coverage width or area index <52 or 53%, respectively, would suggest a lack of optimum dorsal coverage and possible joint incongruence. Despite the strong correlation identified between the measures of dorsal and lateral AFH coverage, the authors suggest considering both techniques to evaluate the overall AFH coverage during screening protocol. Acetabular index angle above 9° is expected to be consistent with hip dysplasia. Coxa-vara was consistent with moderately and severely dysplastic coxofemoral joints. The reported modified FCI hip scoring protocol may bring an improvement on the current situation in Labrador Retrievers; however, further investigation is warranted to prove this possible improvement.

## Data Availability Statement

The original contributions presented in the study are included in the article/Supplementary Material, further inquiries can be directed to the corresponding authors.

## Ethics Statement

Ethical review and approval was not required for the animal study because the retrospective study was based solely on reviews of medical records, including radiographic images, generated during routine veterinary care of the dogs included in the study. Written informed consent was obtained from the owners for the participation of their animals in this study.

## Author Contributions

AM, MN, and CB developed the measurement techniques, approved the selected radiographs, performed data collection and analysis, and drafted the manuscript. CB supervised and approved all aspects of the research and gave the final approval of the manuscript to be published. KA participated in the coordination of data collection, reviewed the manuscript, and gave the final approval of the version to be published. All authors provided the original conception of the project and participated in the research design and read and approved the final version of the manuscript prior to submission.

## Conflict of Interest

The authors declare that the research was conducted in the absence of any commercial or financial relationships that could be construed as a potential conflict of interest.

## Publisher's Note

All claims expressed in this article are solely those of the authors and do not necessarily represent those of their affiliated organizations, or those of the publisher, the editors and the reviewers. Any product that may be evaluated in this article, or claim that may be made by its manufacturer, is not guaranteed or endorsed by the publisher.
